# Aluminate-Based Nanostructured Luminescent Materials: Design of Processing and Functional Properties

**DOI:** 10.3390/ma14164591

**Published:** 2021-08-16

**Authors:** Rocío Estefanía Rojas-Hernandez, Fernando Rubio-Marcos, José Francisco Fernandez, Irina Hussainova

**Affiliations:** 1 Department of Mechanical and Industrial Engineering, Tallinn University of Technology, Ehitajate 5, 19180 Tallinn, Estonia; irina.hussainova@taltech.ee; 2 Electroceramic Department, Instituto de Cerámica y Vidrio, CSIC, Kelsen 5, 28049 Madrid, Spain; frmarcos@icv.csic.es (F.R.-M.); jfernandez@icv.csic.es (J.F.F.); 3 Escuela Politécnica Superior, Universidad Antonio de Nebrija, C/Pirineos, 55, 28040 Madrid, Spain

**Keywords:** ceramic luminescent materials, nanostructure, screen printing, molten salts, NIR emission, down-conversion, nanofibers, sol-gel

## Abstract

Interest in luminescent materials has been continuously growing for several decades, looking for the development of new systems with optimized optical properties. Nowadays, research has been focused on the development of materials that satisfy specific market requirements in optoelectronics, radioelectronics, aerospace, bio-sensing, pigment applications, etc. Despite the fact that several efforts have made in the synthesis of organic luminescent materials, their poor stability under light exposure limits their use. Hence, luminescent materials based on inorganic phosphors are considered a mature topic. Within this subject, glass, glass-ceramics and ceramics have had great technological relevance, depending on the final applications. Supposing that luminescent materials are able to withstand high temperatures, have a high strength and, simultaneously, possess high stability, ceramics may be considered promising candidates to demonstrate required performance. In an ongoing effort to find a suitable synthesis method for their processing, some routes to develop nanostructured luminescent materials are addressed in this review paper. Several ceramic families that show luminescence have been intensively studied in the last few decades. Here, we demonstrate the synthesis of particles based on aluminate using the methods of sol-gel or molten salts and the production of thin films using screen printing assisted by a molten salt flux. The goal of this review is to identify potential methods to tailor the micro-nanostructure and to tune both the emission and excitation properties, focusing on emerging strategies that can be easily transferred to an industrial scale. Major challenges, opportunities, and directions of future research are specified.

## 1. Introduction

Recently, a large number of inorganic, organic, or inorganic-organic luminescent hybrid materials have been extensively explored for optical, opto-electronical, and biological applications. Nowadays, a wide variety of luminescent materials are represented in the forms of glass, glass-ceramics, and ceramics. Here, we are focusing on luminescent materials composed of an inorganic matrix, usually known as a host, and activators or dopants, which are included in the matrix to act as an emitter or a trap. In general, the dopants are rare earth elements, and, to a lesser extent, transition metals, such as V^3+^, Cu^2+^, Mn^2+^, Ti^4+^, Sn^2+^, Co^2+^, Bi^3+^ or Pb^2+^ [1]. Despite the increasing demand for rare earth-free luminescent materials, the efficiency is still quite low [2].

Glasses are quite versatile and are mainly used for lasers, optical fibers and amplifiers due to their high optical transparency. Usually, a low phonon matrix is selected to get rid of non-radiative relaxation. Glass phosphors’ shortcomings are related to their inefficient performance as compared to crystalline counterparts [3]. Moreover, as bulk active materials, their manufacturing is economical and less tedious, providing the freedom to obtain different shapes, sizes, and homogeneity, avoiding optical losses.

On the other hand, glass-ceramics are constituted by crystallites that are uniformly dispersed or embedded into an amorphous glass matrix. Their optical properties are modulated by the nature of the crystallites that can be either on the micron or nano scale, the glass matrix and the interfaces between the constituents [4,5]. The refractive index and morphological differences between the crystal and glass components are considered as the main reasons for scattering losses. This encourages the scientific community to closely match the refractive index of the crystalline phase to the glass-matrix one. Design and fabrication methods are challenging tasks. Despite significant advances in the theoretical understanding of glass structures [6], predictive methods are still far from maturity.

Not only should the optical properties of luminescent materials be improved, the materials themselves should fulfil other requirements, such as having a suitable hardness, fracture toughness, and high temperature stability. For this reason, research in polycrystalline ceramic materials has been growing in parallel with the development of glass-based materials. Luminescent materials that withstand high temperatures, harsh chemical environments, electromagnetic fields, and radiation are highly demanded. These requests have led to the study of ceramic luminescent materials, which have customized spectral properties and decay kinetics.

In general, luminescent materials, as well as ceramics, have been mostly manufacturing in a powder form. For specific applications, luminescent particles are incorporated to form thick or thin films. However, efficient emission of light is limited due to the strong absorption and scattering of the particles. This, alongside the drawbacks regarding thermal stability and aging degradation of the particles embedded in a polymer matrix, have directly led to the development of films, where the volume effect greatly increases the luminescent properties.

Several fabrication methods have been adapted to synthesize ceramic luminescent materials based on conventional approaches, such as solid-state methods and, to a lesser extent, others routes, such as the precipitation method, a sol-gel route [7,8], hydrothermal synthesis [9], laser synthesis [10], and combustion synthesis [11,12,13,14]. Sol-gel derived powders usually accept higher contents of dopants due to their solubility and achieve a better dispersion in the matrix compared to other synthesis methods, in which the quenching concentrations are lower.

Generally, ceramic luminescent materials can be classified based on their chemical compositions. Within the oxide family, Al_2_O_3_ has mainly been studied due to its high performance within engineering ceramics. Specifically, the α-alumina phase has been selected due to its stability and wide availability. Both undoped and doped alumina compounds show luminescence. Undoped alumina represents a high potential for use in dosimeters [15] due to the high concentration of oxygen vacancies, which produces F-centers and the photo and cathodoluminescence in the UV region derived from the F-centers. Regarding doped alumina, Cr-doped Al_2_O_3_ powders have been the most studied; the luminescence response has been found to be similar to ruby crystal, giving a framework to replace single crystals by employing polycrystalline alumina with a reduced cost and with a greater versatility of shapes and dimensions [16].

Garnets are also well-known as a host family for optical applications; these compounds are based on the combination of a rare earth oxide and a metal oxide, the most representative materials of this group are Y_3_Al_5_O_12_ (YAG) and Y_3_Ga_5_O_12_ (YGG). YAG may be doped with Eu [17], Ce [18], or Nd [19,20] to enhance performance. It is important to highlight that Nd-YAG is one of the most important laser crystals for generating a 1.06-μm NIR emission. The primary reason for the interest in aluminate-based luminescent materials is its efficient luminescence response compared to other families, such as oxides. The undistorted and distorted structures formed by rings of AlO_4_ tetrahedra and AlO_6_ octahedra provide lattice conditions for the ease incorporation of activators that provide the luminescence properties to the end product. In addition, some inherent defects, such as vacancies and structure inversion contributing to the creation of anti-site defects, give added value into their luminescence response.

Another remarkable family of luminescent materials is the sulfides. Among them, calcium sulfides (CaS) doped with Eu^2+^, Tm^3+^, and Ce^+^, or with Bi^3+^, show emission in red and blue lights [21,22]. Sulfide development has been in parallel with that of LEDs; the first white LEDs were based on a blue LED joined with a sulfide phosphor. However, sulfides are sensitive to moisture and thermal quenching. For this reason, the research on aluminate-based phosphors has gained enormous attention. Generally speaking, metal aluminates doped with RE demonstrate a suitable luminescence performance due to the intrinsic properties of spinel or trydimite structures. This is the case for the alkaline earth aluminate MAl_2_O_4_ (where M = Ca, Sr, Ba) and also alkaline earth hexa-aluminates related to magnetoplumbite and β-alumina.

Until now, the number of types of phosphors has increased significantly, and there are new modified hosts and dopants from transitions metals and rare earths that open up a chart of phosphors. Depending on the emission, excitation, and end-product response, a huge list of appropriate phosphors for each field is already available. In this review, some phosphors based on alkaline earth aluminates and metal aluminates are reported. Our work is focused on materials that display luminescence and persistence luminescence. Mostly, RE have been used as activators, achieving emissions in the visible and NIR ranges.

Taking into consideration the current phosphor market, there are still some limitations that hinder their use in practical applications. In the case of phosphors in a powdered form, commercial phosphor particles in the micron size range, which have been mainly produced using conventional solid-state reaction processes, have shortcomings related to their agglomeration and irregular shapes and sizes. Nano-scale phosphors could be a promising alternative due to the confinement effects; however, their low stability, together with complex synthesis routes, restrict their use. As luminescence is affected by the morphology and size of particles, many efforts have been made to synthesize well-defined morphologies and sizes. To solve the problem of agglomeration, hydrothermal, microemulsion and sol-gel methods have been successfully exploited; small particles at low temperatures can be synthesized, but the formation of a single phase should be optimized [23]. Therefore, combining improvements in efficiency and stability of phosphors is still required; the scientific community is looking for new strategies to provide a framework for the design of the next generation of phosphors based on nano-architectures, increasing performance and, at the same time, wavelength tunability.

## 2. Designing Strategies for Aluminates-Based Luminescent Materials

Luminescent nanostructured materials have drawn the interest of the scientific community due to their optical and chemical properties, which show better a response in comparison with their bulk counterparts. Their design size, morphology, and phase content have led to a surge of new synthesis methods. Chemical routes, such as hydrothermal, co-precipitation, micro emulsion, solvothermal, combustion, sol-gel methods, and microwave-assisted reactions, are usually used to produce nanomaterials, and are designated as bottom-up approaches. Ceramic powders with ultrafine grains and spherical morphologies are commonly obtained via microemulsion, in which the growth of particles is limited by micelles. Solvo-thermal or microwave assisted bottom-up processes allow to control the size of phosphor particles through surfactant or chelating agents. Nevertheless, the complicated fabrication procedures limit the practical applications of these nanomaterial solutions, which do not require sophisticated processing in order to have easy scale-up. Through the use of top-down approaches, such as high- or low-energy milling processes, nanophosphors can also be produced. However, the milling process deteriorates optical properties due to the large numbers of defects on the surface and the lower crystallinity of the obtained non-spherical phosphors.

### 2.1. Luminescent Materials by Molten Salts Assisted Process

Looking for a synthesis method that can meet all requirements, a molten salts strategy has been used for the synthesis of nanostructured luminescent materials to be transferred to an industrial level. Not many phosphors have been obtained using a molten salt-assisted route. The first works on phosphors made using molten salts date to the last decade; Lei et al. [24] obtained sphere-like and rod-like Gd_2_MoO_6_:Eu^3+^ phosphors depending the used flux (NaCl or KCl). The same authors obtained a red phosphor based on ZnWO_4_:Eu^3+^ [25] using LiNO_3_, NaNO_3_, and KNO_3_ as a flux. Y_2_O_3_:Eu, Bi was synthesized for the first time by Wu et al. [26]; the red emitter had an octahedral morphology that was obtained using a KNO_3_–NaNO_3_ eutectic mixture. Cerium-activated Y_3-x_Ce_x_Al_5_O_12_ was first synthesized using molten salts with NaNO_2_-KNO_2_ and NaCl-KCl [27]; the morphology could be tuned from spherical-like to cubic shaped using different salts. Moreover, YAG doped with cerium non-aggregated particles with a spherical morphology were obtained using mixtures of Na_2_SO_4_-BaF_2_ as the molten salt [28]. The salt ratios modulated the diameter of the spherical particles, which shows peak emission at 535 nm [28]. Other quasi-spherical Y_2_O_3_:Eu^3+^ particles were obtained using Na_2_CO_3_, S, and NaCl salts. The particles synthesized with molten salts allowed to improve the luminescence intensity by 30% in comparison with particles obtained without salts [29]. Recently, red-emitting MgAl_2_O_4_:Mn^4+^ single-crystal phosphors have been produced using LiCl as a molten flux [30].

**Figure 1:**
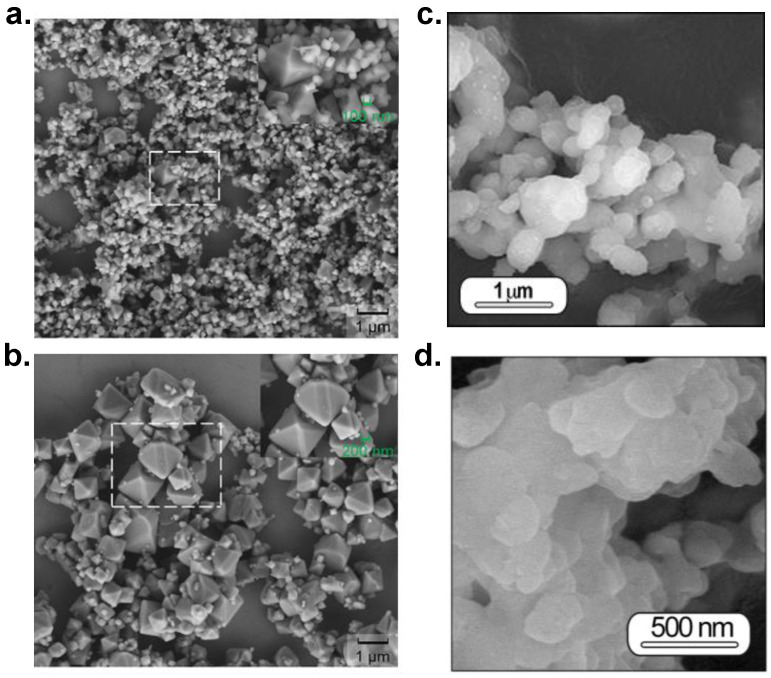
SEM micrographs of MgAl_2_O_4_:Mn phosphors heated at 950 °C for 6 h in 90N_2_-10H_2_ atmosphere using (**a**) nano α-Al_2_O_3_ and (**b**) nano γ-Al_2_O_3_. Reprinted with permission from [30]. Copyright 2020, *American Chemical Society Publishing*. FE-SEM micrographs of powders based on SrAl_2_O_4_:Eu, Dy heated at 1000 °C for 2 h in 90N_2_-10H_2_ atmosphere, employing a salt/SrAl_2_O_4_ molar ratio of 3:1 using (**c**) nano α-Al_2_O_3_ and (**d**) nano γ-Al_2_O_3._

The idea behind molten salt synthesis is to promote mass transfer and transport using a liquid media at the synthesis temperature. The nature of the salt greatly effects the chemical reactivity of the precursor used; depending on the composition a relatively low melting point can be achieved. Generally, nitrates, hydroxides, chlorides, and sulfates are employed; individual ionic salts or their corresponding eutectic mixtures are selected, and the advantage of mixtures are that they endow a low melting point and usually a reduced viscosity. The morphology, size, and nature of the precursors used govern the growth process of a template formation, a dissolution-precipitation process, or a mixture of both. When both reactants are soluble in salt, the dissolution-precipitation mechanism controls the growth. On the contrary, if one of the reactants is less soluble that the other, the dissolution-diffusion mechanism (known as a template formation or template growth) occurs; the soluble reactant is dissolved in the initial stage and then diffuses onto the surface of the less-soluble precursor. Figure 1 shows the morphology of two aluminates, magnesium and strontium aluminate phosphor materials prepared by molten salts employing two different nanosized, alpha (α-Al_2_O_3_) and gamma (γ-Al_2_O_3_), aluminas and MgO and SrCO_3_ particles in the micron range. Here, the dissolution-precipitation tends to dominate the growth mechanism. As the aluminates formed tend to retain the original nano γ or α-Al_2_O_3_ shape, the authors suggested the occurrence of a mix mechanism (dissolution–diffusion–precipitation process), governed by the dissolution-precipitation process. When γ-Al_2_O_3_ is used, Mg^2+^ or Sr^2+^ diffusion around the nano γ-Al_2_O_3_ is higher in comparison to the alpha phase, and the end aluminate is notably large. Raw α-Al_2_O_3_ promotes template mechanism preserving the size of the precursor of the alpha alumina more, while the raw γ-Al_2_O_3_ stimulates the dissolution-precipitation process. Despite there the fact that there is a mixing mechanism in both cases, the prevailing mechanism defines the end size of the aluminate.

The synthesis of magnesium aluminate MgAl_2_O_4_:Mn^4+^ was done using ionic salts, such as LiCl, NaCl, and KCl. As the melting point of LiCl is low, this salt was the one that accelerated synthesis, avoiding residual MgO. On the other hand, SrAl_2_O_4_:Eu, Dy was synthesized using a NaCl-KCl eutectic mixture that has a melting point around 659 °C, which is lower than the individual ionic salts, with melting points at 903 and 954 °C for NaCl and KCl, respectively. To the best of our knowledge, our research group was the first to develop persistent phosphors using the molten salts route [31].

**Figure 2:**
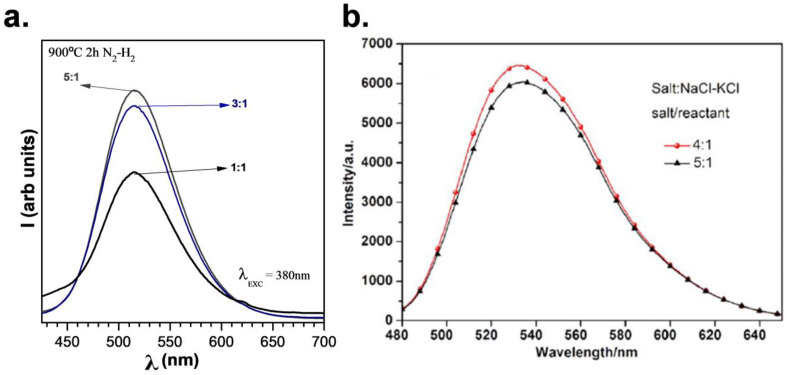
(**a**) Photoluminescence emission spectra as a function of the salt/SrAl_2_O_4_ molar ratio of 1:1, 3:1, and 5:1 of SrAl_2_O_4_:Eu, Dy phosphor synthesized at 900 °C. (**b**) Emission spectra of Ce^3+^ doped yttrium aluminum garnet using salt-to-reactant molar ratios of 4:1 and 5:1. Reprinted with permission from [34]. Copyright 2019, IOP Publishing, Ltd.

In our previous works [31,32,33], the correlation of the amount of salt and photoluminescence intensity was studied thoroughly. In the case of employing SrAl_2_O_4_:Eu, Dy as precursor of nano α-Al_2_O_3_, an increase in the emission intensity was observed when the salt/SrAl_2_O_4_ molar ratio increased from 1:1, 3:1, to 5:1. The emission increment is attributed to the different percentages of monoclinic and hexagonal polymorphs, using different salt ratios (Figure 2a). A similar behavior has been observed when YAG: Ce was synthesized using NaCl-KCl (Figure 2b), it was found that the emission intensity showed a rising tendency as the ratio of salt increased to 4:1; using a higher salts ratio, such as 5:1, the intensity decreased slightly [34].

**Figure 3:**
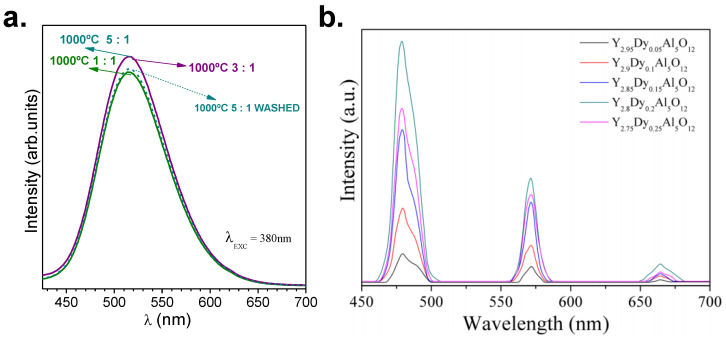
(**a**) Photoluminescence emission spectra as a function of the salt/SrAl_2_O_4_ molar ratio of 1:1, 3:1 and 5:1 of SrAl_2_O_4_:Eu, Dy phosphor synthesized at 1000 °C before and after washing. (**b**) The emission spectra of Dydoped yttrium aluminum garnet using different Dy concentrations, after washing 12 times. Reprinted with permission from [36]. Copyright 2019, John Wiley and Sons.

It is fundamental to emphasize that one of the requirements for the selected salt is related to its easy elimination, either through evaporation during synthesis or by washing in a further step. In most cases, during synthesis, the salt is eliminated because the used temperature usually exceeds the melting point. In case of mixtures of salts, higher temperatures should be used due to the possible vaporization of individual salts. Some grains of individual salts can remain unreacted in the mixture, so leftovers or excess of salts interact well with the phosphor material. If the temperatures of the process do not promote the evaporation of the salts, a washing step is required. In principle, almost all salts can be eliminated by washing out with water or other polar solvents [35]; nevertheless, some phosphors are not resistant or their luminescence response decreases by exposure to water. This shortcoming can be overcome by washing with other media. Here, both approaches are addressed; the phosphors based on strontium aluminate were shed with glycerin and those based on yttrium aluminate with water. Figure 3 shows the drop in luminescence when a washing cycle is performed for phosphors based on strontium aluminate, employing a salt ratio of 5:1 (eutectic mixture NaCl-KCl).

The washing step was carried out using glycerin, avoiding a hydrolysis reaction, due to the sensitivity of strontium aluminates to water. For salt ratios of 3:1 and 1:1, the washing step can be skipped because there is no salt remaining in the end product. Figure 3b shows the emission of Y_3-x_Dy_x_Al_5_O_12_ (x = 0.05, 0.10, 0.15, 0.20, and 0.25) phosphors, employing NaCl-KCl molten salt (ratio of the initial reagents and the NaCl–KCl mixture: 1:4). The YAG:Dy phosphor was fired at 1100 °C and washed with deionized water at least 12 times [36]. Both washing approaches were successfully applied, suggesting that the removal of the remaining salts was not an issue with the use of this synthesis route.

**Figure 4:**
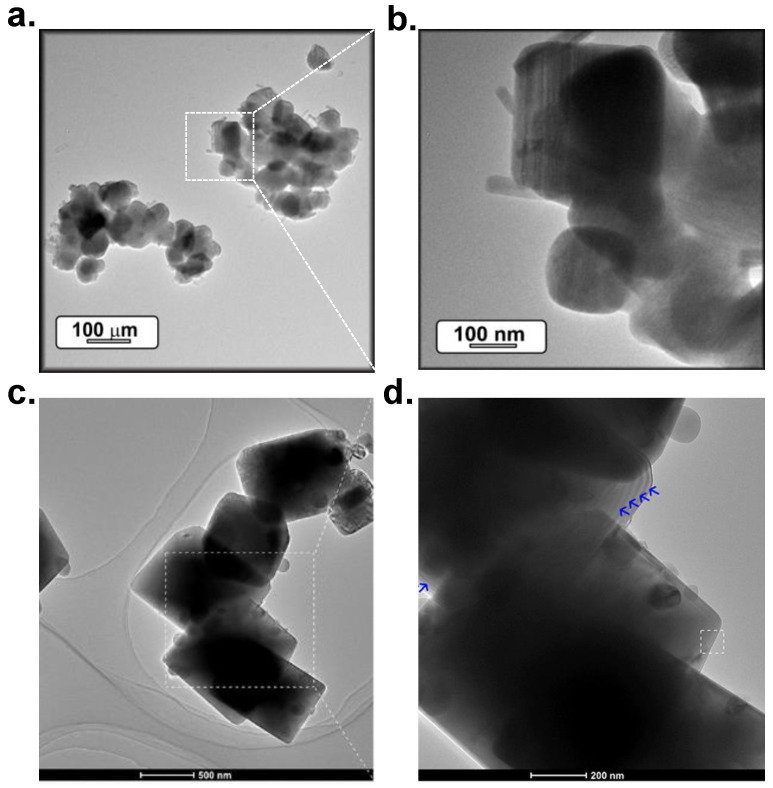
(**a**,**b**) Low- and high-magnification TEM characterization of particles based on SrAl_2_O_4_:Eu, Dy synthesized at 900 °C. (**c**,**d**) TEM images of particles based on MgAl_2_O_4_:Mn^4+^. Reprinted with permission from [30]. Copyright 2020, American Chemical Society Publishing.

In addition, the molten salts approach provides conditions to obtain particles with a high crystallinity, smooth surface, and texturation. Figure 4a–d exhibits TEM images of zinc aluminate and magnesium aluminate particles; in both cases a semi-rectangular shape is observed. The particles of high-crystallinity are interconnected forming agglomerates of less than 200 nm in size. Generally, a better crystallinity means a fewer number of defects and a stronger luminescence, which are crucial aspects in phosphor performance.

A closer examination of the particle morphology indicates the presence of a rod-like structures on the edge of the equiaxed particles, as shown in Figure 4b–d. In the case of strontium aluminates, these rod-like structures can be attributed to Sr_2_Al_3_O_6_ particles, promoted by local excess of SrCO_3_.

### 2.2. Promoting Template-Mechanism by Molten Salts Assisted Process

**Figure 5:**
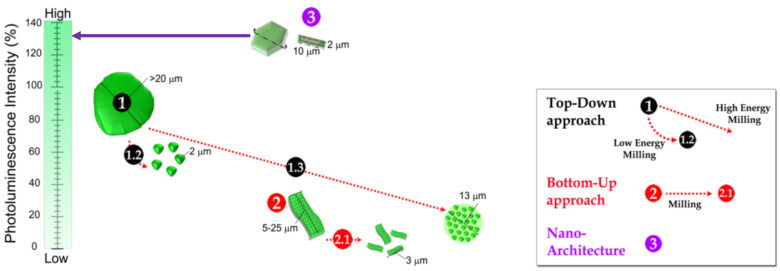
Scheme of the % of photoluminescence intensity, taking as reference commercial powder as a function of the morphology and particle size for SrAl_2_O_4_:Eu, Dy particles synthesized by top-down, bottom-up, and nano-architecture approaches.

The synthesis of particles by molten salt synthesis is governed by two main mechanisms: dissolution–precipitation and dissolution-diffusion. The synthesis of nanophosphors is commonly done using dissolution–precipitation, where the molten salt-assisted process is conceived as a bottom-up approach. Conventionally, the employment of bottom up approaches implies a lower luminescence intensity for nanophosphors obtained in comparison with a micro-sized counterpart. Top-down approaches have similar tendencies due to the defects generated on the surface. A synergy between micro and nano concepts can be conceived when a nano-architecture is developed. In the case of phosphors, for the specific applications, platelet-like shapes are interesting, because one of the dimensions is below ≤2 µm. Using the molten salt strategy, it is possible to design a scalable process of self-supported nanostructures onto microscale particles as fragments of a puzzle to improve the performance of phosphorescent materials, paying special attention to rare earth scarcity. This concept was developed in our previous work for strontium and calcium aluminates. In particular for the case of SrAl_2_O_4_:Eu, Dy, a scheme that depicts the photoluminescence features of particles as a function of the synthesis is shown in Figure 5. A similar picture can be translated for other phosphors based on aluminates.

**Figure 6:**
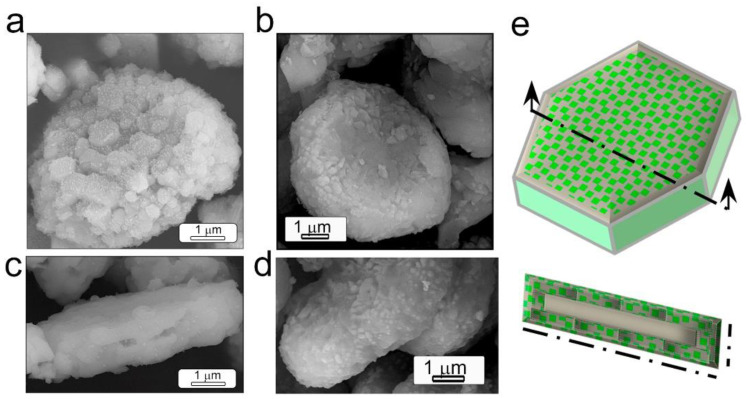
(**a**–**d**) Field-emission scanning electron microscopy (FE-SEM) micrographs of the synthesized particles based on SrAl_2_O_4_:Eu, Dy and CaAl_2_O_4_:Eu, Nd. (**e**) Scheme of the surface and cross-section of the nanostructures obtained with a platelet-like morphology.

In the scheme (Figure 5), a comparison of SrAl_2_O_4_ particles obtained using top-down, bottom-up, and molten salts strategies is shown. Using a conventional method, for example solid state reaction, irregular particles with >20 μm particle sizes and >100 nm crystallite sizes are obtained. These particles are considered as the commercial reference, and their emission signaled a reference for photoluminescence intensity (we use 100% of PL signal). If a top-down strategy, such as milling, is carried out to decrease the particle size, it is possible to obtain well-distributed small particle sizes, but the as-obtained phosphor showed a much lower photoluminescence intensity and a shorter persistent time compared to the corresponding reference material. Using low-energy dry milling (LDM), designated as 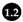 in Figure 5, the obtained average size is 2.8 μm. The advantage of this method is particle dispersion during the milling process. This process generates defects at the particle surface. Using high-energy dry milling (HDM designated as 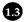 in Figure 5), a broad particle size distribution was obtained due to the agglomeration state, and the process significantly damages the phosphor structure. Therefore, the solution could be the development of traditional approaches for the synthesis of nano-sized SrAl_2_O_4_:Eu^2+^, Dy^3+^ material, called bottom-up methods. One strategy is combustion synthesis, indicated as 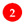 in Figure 5, resulting in nanostructured flakes with sizes ~5–25 μm in diameter and ≤1 μm in thickness. Therefore, a top-down strategy can be used; the material is milled using a dry milling process, marked as 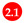 in Figure 5, obtaining average particle sizes. However, the photoluminescence response decreases. The approaches of sol-gel, hydrothermal, and microemulsion synthesis have also been evaluated for powders with a lower photoluminescence intensity. Moreover, there are other disadvantages, including multistep procedures and post-thermal treatments, that increase processing costs. For this reason, a nano-architecture strategy, specified as 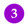 in Figure 5, was developed to synthesize particles with a required performance. Following an overview of principles, which guide nanocrystal formation, the emerging design criteria are outlined for shape-controlled nanocrystals prepared using molten salt synthesis, a synthetic strategy that provides control over crystal growth. Employing less-reactive Al_2_O_3 _and particles with a larger size, a well-defined morphology has been developed with the help of a template-assisted technique, where nanostructures are self-supported in alumina’s core. Figure 6 evidences that particles preserve the platelet-like morphology of the α-alumina micro-particles. The template mechanism dominates the reaction path for both systemscalcium (Figure 6a) and strontium aluminate (Figure 6b). The dissolution of strontium and calcium carbonate precursor takes precedence, and the molten salt flux transports them to the surface of Al_2_O_3_. The average thickness of synthesized aluminate phosphor was 2 µm. In addition to the morphology, the synthesized phosphors were characterized by a core-shell structure, in which the shell was composed of nanostructured phosphor and the core was unreacted alumina. The processing of these core-shell structures makes possible a reduction in incorporated rare earths by half. Figure 6e depicts the scheme of the core-shell structures obtained. The nanostructured microparticles possess the advantage of a highly reflective core and a thickness of the shell that is sufficient to absorb the excitation photons and maximize the phosphor efficiency.

**Figure 7:**
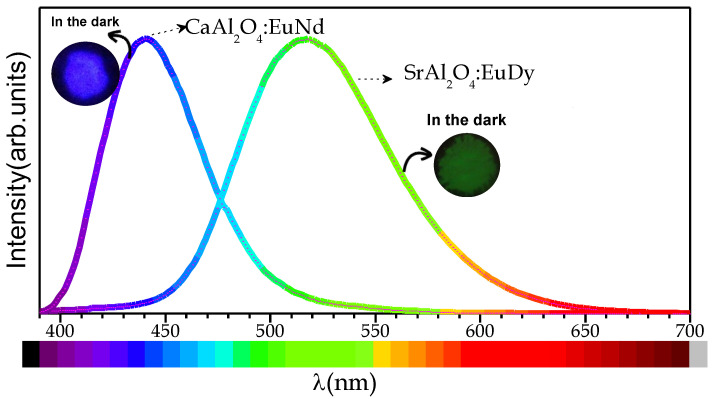
Photoluminescence emission spectrum (λ_EXC_ = 365 nm) of SrAl_2_O_4_:Eu Dy and CaAl_2_O_4_:Eu Nd platelet-like particles.

Depending on the host matrix and the dopants incorporated, it is possible to tune the emission wavelength. Figure 7 shows the emission ranging from blue to green for the particles based on CaAl_2_O_4_:Eu, Nd and SrAl_2_O_4_:Eu, Dy, respectively, under 365 nm excitation. The persistent luminescence is demonstrated in both systems; the insets of the particles after being activated for 10 min in the dark shows that, after the excitation is cut off, afterglow remains.

Previously, nanomaterials solutions based on the development of nano-architectures supported by platelet and spherical-like shape morphologies have been described. The framework stability and network-structured phosphors contribute to the development of phosphors with a high optical performance. Nevertheless, fiber morphology has been not addressed by the molten salt route. The next section is devoted to luminescent aluminate materials with nanofiber shapes. Nanofiber morphology may be a promising direction for luminescent applications that focuses on large surface-to-volume ratios. More straightforward approaches for fibers synthesis are solution-based strategies, such as the sol-gel method.

### 2.3. Luminescent Materials Obtained by Sol-Gel Synthesis

Most ceramics fibers are manufactured using an electrospinning route. The main advantage of this technique is the high aspect ratio of the obtained fibers; the method is quite versatile and the morphology is tuned by the spinneret. However, it is difficult to optimize the chemistry and it is not always easy to find the proper combination of ceramic precursor, polymer, and solvent. Controlling the diameter and morphology is still not a trivial task. Regarding alkaline earth aluminates, CaAl_2_O_4_:Eu^2+^, Nd^3+^ has been produced using a modified electrospinning process [37], during which a carbon shell is developed as a result of the PVP and organic solvent decomposition and carbonization. After annealing, this carbon shell is eliminated and fibers of 160 nm in diameter remain. The fibers exhibit the emission at 445 nm under 345 nm excitation.

Recently, SrAl_2_O_4_: Eu/Yb nanofibers around 2 µm in length and 400 nm in diameter were synthesized [38]. The fibers exhibited up and down conversion luminescence under excitation at 478 and 365 nm, respectively. Usually, fibers produced by electrospinning or modified sol-gel electrospinning processes are long fibers. Short fibers are also required, so a direct synthesis process is highly desired. Long fibers can be cut, but this operation always adds additional steps to the manufacturing process. Following, a similar reaction mechanism to that which happens in molten salt synthesis, the impregnation generated using a sol-gel process can be useful to obtain aluminates if the initial fibers can serve as a source of alumina. Using this method, the dimensions of the final fibers will be modulated depending the nature and morphology of the template precursor.

**Figure 8:**
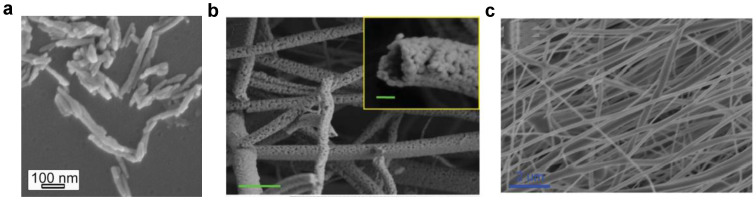
Field-emission scanning electron microscopy (FE-SEM) micrographs of the synthesized nanofibers based on (**a**) ZnAl_2_O_4_:Nd, Ce, (**b**) ZnAl_2_O_4_:Cr. Reprinted with permission from [40]. Copyright 2012, Royal Society of Chemistry Publishing. And (**c**) ZnAl_2_O_4_:Nd. Reprinted with permission from [41]. Copyright 2013, American Scientific Publishers.

This approach has been developed for the synthesis of zinc aluminate materials. Ceramic nanofibers based on ZnAl_2_O_4_:Ce, Nd with an average diameter of 50 nm have been obtained using sol-gel synthesis. Figure 8 shows the morphology of ZnAl_2_O_4_: nanofibers co-doped with Ce and Nd [39] (Figure 8a) and doped with only Cr [40] (Figure 8b) and Nd [41] (Figure 8c) synthesized using sol-gel and electrospinning, respectively. The surface of the nanofibers co-doped with Ce and Nd obtained using sol-gel after annealing at 1200 °C is smooth; a similar morphology is demonstrated for fibers only doped with Nd obtained by electrospinning. However, the surface of the nanofibers doped with Cr obtained using a single-nozzle electrospinning process become rough after calcination at 1200 °C. In the case of sol-gel synthesis, shorter strands were produced due to impregnation using the sonication process, as compared with the long fibers obtained by the electrospinning method.

**Figure 9:**
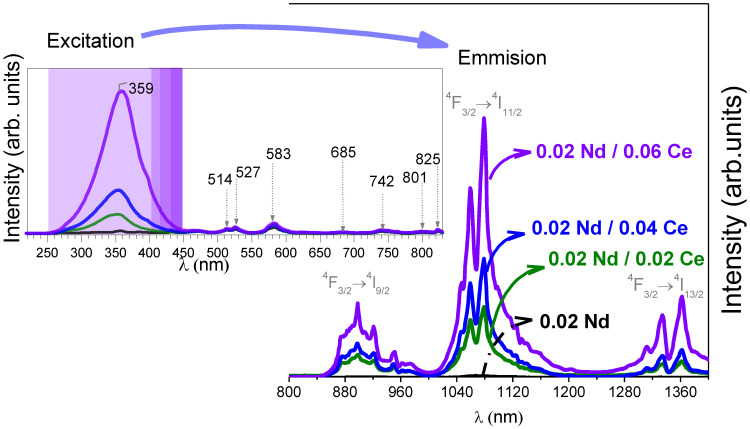
Excitation and emission spectra of the synthesized nanofibers based on ZnAl_2_O_4_:Nd, Ce.

Figure 9 shows the emission and excitation spectra of the ZnAl_2_O_4_ nanofibers doped with Nd and co-doped with Ce, fixing the Nd content to 0.02 (%mol) and with different Ce contents ranging from 0.02 to 0.06 (%mol). The modulation of the concentration of dopants also has an influence on the emission intensity. The presence of cerium boosts the photoluminescence emission due to a down-conversion process through the Ce-Nd energy transfer band.

The progress on these materials opens as well a section of the portfolio of biocompatible luminescent materials. Both ZnAl_2_O_4_: nanofibers doped with Nd and co-doped with Ce and Nd exhibit NIR emissions in the optical window for cells and tissues, opening their use in biological markers. For both systems, the cytotoxicity and viability of cells were evaluated.

**Figure 10:**
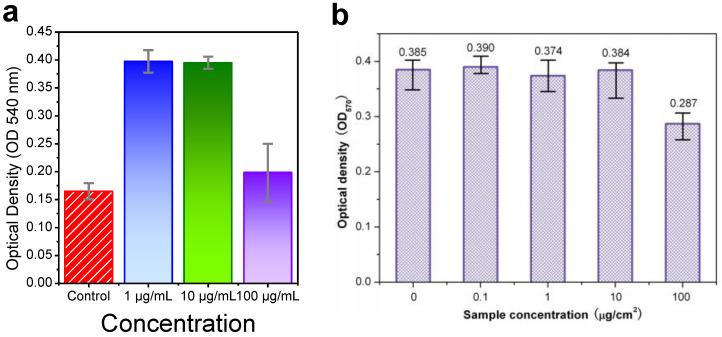
Evaluation of cytotoxicity. (**a**) MTT assay-the HeLa cells were incubated with a 1, 10, and 100 µg/mL concentrations of ZnAl_2_O_4_ for 24 h, and a control was also evaluated for ZnAl_2_O_4_: nanofibers doped with Nd and co-doped with Ce. (**b**) Cytotoxicity among the various concentrations 1–100 µg/cm^−2^. Reprinted with permission from [41]. Copyright 2013, American Scientific Publishers.

To determine cell viability, MTT assays are usually performed. In the case of the ZnAl_2_O_4_: nanofibers doped with Nd and co-doped with Ce obtained using sol-gel synthesis, HeLa cells were employed. On the other hand, for ZnAl_2_O_4_: fibers doped only with Nd obtained by electrospinning, BMSCs were used as model human cells. Figure 10a,b exhibits the optical density (OD) result of the absorption band located at 540 and 570 nm. The OD is higher than the blank (control sample) for all concentration from for 1 and 100 µg/mL for the nanofibers co-doped. For the fibers only doped with Nd obtained by electrospinning route, no big difference is observed for concentrations up to 10 µg/cm^2.^ However, with higher concentrations, 100 µg/cm^2^, the viability decreases up to 75%. In any case, for both systems obtained by different synthesis routes, there is no cytoxicity activity for the materials based on zinc aluminate doped with Nd and Ce. These results demonstrate the potential of use these nanofibers for biological markers and cell imaging.

The luminescent materials in the form of films have also attracted a great deal of attention; for example, in the development of advanced LEDs or sensing materials. In the case of LEDs, the replacement of traditional encapsulation technology can help to acquire a higher thermal stability and heat dissipation. The main advantage of luminescent films is that they can become a straightforward part of a device format. The main drawback of film synthesis is that synthetic efforts start to be more versatile. Here, we will describe, as a selected example, the fabrication of luminescent films based on aluminates using a cost-efficient and flexible strategy, i.e., modified screen printing assisted by molten salts.

### 2.4. Designing Strategies for Ceramic Luminescent Films by Modified Screen Printing Assisted by Molten Salts

There are two main strategies to fabricate luminescent films, indirect and direct strategies. With the indirect strategies, luminescent particles are incorporated into a matrix to form thick or thin films. Generally, a polymer matrix is employed; the main drawback is related to thermal stability and aging degradation of the luminescent particles embedded in the polymer matrix. Other indirect approaches rely on the dispersion of phosphor particles within a bulk glassy matrix (particles into the glass, PiG) and by partial glass crystallization, GC. In both approaches, the selection of glass composition is of fundamental importance to obtain a suitable glass network. The glass network should neither react with particles embedded, nor crystallize, and should have a similar refractive index with the phosphors in the case of the PiG approach. The main drawback is that the particles are often not homogeneously dispersed. The GC approach faces problems related to the incorporation of activators in the crystalline phases and the heterogeneous nucleation process; usually, nucleation only happens on the surface. In direct strategies, the films are directly produced either using sol-gel using dip-casting or spin-coating techniques or by the employment of more sophisticated methods, such as sputtering, ion beam evaporation, ultrasonic spray pyrolysis, and pulsed laser deposition. In terms of methodological simplicity and cost-effectiveness, the synthesis of films using solution-based processes, such as sol-gel deposition with dip or spin-coating, are the most extensively employed. However, there are still some limitations related to scaling-up of this technology and the difficulty in obtaining thick layers, unless the deposition of multiple layers is done by repeated the whole process. The shrinkage of the gel and initial density are the main factors that have an influence on the final thickness [42]. Moreover, the structure of the gel promotes or hinders crack nucleation; therefore, depending on the chemical composition, the correlation between the experimental conditions (time, speed, solvent content, catalyst, substrates, annealing, hydrolysis, and condensation), the film thickness, and the phase composition, requires in depth study.

Different luminescent aluminates have been developed in the form of films. ZnAl_2_O_4_ films have been doped with Eu, Tb, or Tm using an ultrasonic spray pyrolysis technique [43,44]; films doped with Mn have been produced using a sol-gel route [45]. Concerning strontium aluminates, a SrAl_2_O_4_ polymorph has been obtained by sputtering, ion beam evaporation, ultrasonic spray pyrolysis, and pulsed laser deposition [46,47,48,49,50,51,52].

**Figure 11:**
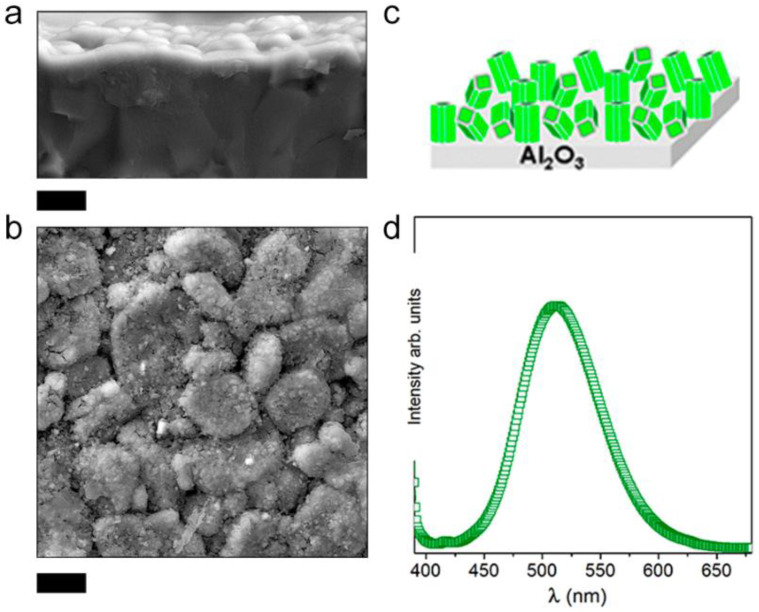
Field-emission scanning electron microscopy (FE-SEM) micrographs of the SrAl_2_O_4_:Eu, Dy, films obtained over polycrystalline alumina substrate: (**a**) cross-section and (**b**) surface view. (**c**) Scheme of the growth of the film over a polycrystalline substrate. (**d**) Emission spectra of the SrAl_2_O_4_:Eu, Dy, film obtained by screen printing. The scale bar in (**a**,**b**) represents 5 µm.

Keeping in mind the importance of finding a scalable technology, the screen-printing method has been used to obtain luminescent films. The screen-printing technique has been previously exploited to obtain thicker transparent Ce-YAG PiG film with a thickness of >19 μm [53] on a glass substrate. The prepared inks have already dispersed phosphor particles, so the method follows the concept of an indirect strategy [54]. Our group has developed a screen-printing strategy assisted by a molten salt route; the approach is a direct strategy. In principle, the screen-printing technique has been conceived of for thick films, but it is possible to get films below 1 μm using assisted molten salt flux. The employment of a molten salt flux promotes crystal growth in the case of the synthesis of particles; the same concept has been translated to film synthesis. A polycrystalline alumina substrate (not polished) has been used for the deposition of ink that employs a molten salt based on a eutectic NaCl and KCl mixture. From a cross-section view, films with ~750 nm in thickness are crack free (Figure 11a). By employing a non-polished substrate, the coating reveals the hexagonal grains of the polycrystalline substrate (Figure 11b). Taking into account the template formation mechanism, the developed film follows the template of alumina grains. Figure 11c shows a scheme of film growth when a polycrystalline substrate is used. By modulating the nature of the substrate, it is possible to also grow epitaxial films promoting an orientation degree. The crystal texturation of screen-printed films should be highlighted as the oriented films are not usually obtained by a simple synthesis method such as this. A uniform thickness and the desired phase composition imply the suitable incorporation of the dopants to get a functional film and the mass transport process of all precursors, including the activators, should be carefully optimized to promote the formation of the desired growth. The emission spectrum shows that a luminescent film is successfully prepared; green emission upon excitation at 380 nm of synthesized SrAl_2_O_4_:Eu, Dy film is observed.

The optimized screen-printing method assisted by molten salts, which was developed by our group, can be adapted to other aluminates to produce other active materials for other emission and excitation ranges.

## 3. Perspectives and Applications

The main challenge for the luminescent materials research community is to develop strategies that consider further up-scaling and cost-effective production of materials in both powder and film forms. The proposed approaches based on sol-gel and molten salt synthesis exhibit enormous flexibility for different system production. These methodologies are proven to produce materials at the industrial scale for other systems and, thus, anticipate the scalability for nanostructured inorganic phosphors. The ability to tailor engineering nanostructures and at the same time tuning both the emission and the excitation ranges represents a key point for the development of devices for application in printable optoelectronics, energy storage, sensor technology, as well as products in medicine. The development of mass production processes will open new application in cost-efficient applications for common usage; for example, in signage, textile and printing areas. Moreover, incorporation of the luminescent materials into additive manufacturing will open new possibilities beyond aesthetic or design applications.

The use of rare earth (RE) as activators of phosphors represents a relevant limitation. Rare earths are critical materials due to their scarcity, environmental impact and cost. The high demand for RE in functional applications will imply a foreseeable increase for the next few years that will depend on the availability of their supply also conditioned by geopolitical evolution and international trade agreements. The forecast implies an increase in the cost of raw materials that will restrict certain applications. On the one hand, the use of strategies to reduce the use of rare earths by inorganic phosphors is an incentive for researchers. In this sense, nanostructured coatings have shown an increase in the efficiency of the systems that must be enhanced. The recovery of rare earths from industrial waste is a necessary aspect to be addressed in the coming years in order to maximize the use of scarce resources on planet earth. Given that the circular economy approach will be essential in the coming years for industrial product development, the entry of rare earths into circular economy processes requires commendable efforts. The strong dependence of the response of rare earths with minority elements and impurities must be addressed in order to achieve new, more robust and tolerant formulations, aspects that result far from obvious for materials with an optical response. Last but not least, the development of inorganic materials with luminescent properties without rare earths is an emerging field that requires a strong investment in the coming years. Some recent examples in dual micro-nanostructure glass-ceramic feldspar nanocrystals having aluminate tetrahedra in their structure that present a relevant luminescent efficiency without rare-earth activators [55,56]. However, there is still a need to increase its tuning capacity for broadcasts.

Concerning the application of inorganic phosphor as optical probes in biomaging and biosensing, the luminescent powders are more convenient for the efficient penetration cell ability for in vivo and in vitro studies. Nevertheless, the luminescent particles can be functionalized for targeting specific tumor or diseases and furtherly incorporated in an adequate fluid. Despite, the stability should be verified and the regulation and standardization needs further steps, the incorporation of phosphors in powder form is still a solution for multiple bio and lightning applications.

Nowadays, material that can have isotropic lighting characteristics and high mechanical properties is highly desired [57]. Ceramics coatings in the form of films can solve these requests due to the strong mechanical performance combined with an enhanced optical response. In addition, the stability increases the lifetime of the final product as compared to the polymer hybrid composites. The development of transparent films with a controllable photon release is of a great interest for anti-counterfeiting technology and energy storage applications from the VUV to NIR range [58]. In addition, the technology can be applied for not persistent materials, as well. A field that is still under construction is the UV-LEDs, the UV–C emission has been proven as efficient technology for surface disinfection. Therefore, the development of undoped and doped luminescent materials, which emit in the range of 200-280 nm for inactivating some virus and bacteria, is other area in which the aluminates can be used. We also envision that the concept of developing submicron films by screen-printing could be further explored with functionalizing by plasmon deposition on the surface.

## 4. Conclusions

Ceramic luminescent nanomaterials, specifically assembled nano-architectures, demonstrate a better performance in terms of photoluminescence response together with customized microstructures and morphologies compared to bulk counterparts. The methods of assembling particles using sol-gel or molten salts and uniform films using a modified screen printing assisted by molten salts show the greatest potential to develop luminescent materials for a wide range of applications in optical (lighting, photonics, indicators), optoelectronic, bio-sensing, and anti-counterfeiting fields. The synthesis routes presented in detail in this review are able to overcome the disadvantages of a complex synthesis and present beneficial solutions for the scaling-up required for industrial applications. The sol-gel method used to generate nanofibers with large surface-to-volume ratios is of crucial importance for the development of bio-markers. The combination of the screen-printing technique and the assisted process through the use of molten salts shows the potential to control the thickness of uniform films. However, the extensive application of this combined strategy can also be fully realized for other aluminate-based materials. Further efforts are needed to explore the enormous potential of this flexible synthesis to create multifunctional ceramic materials, where the luminescence properties can be exploited.

## Data Availability

Data sharing is not applicable to this article.
